# Long-chain omega-3 polyunsaturated fatty acids are reduced in neonates with substantial brain injury undergoing therapeutic hypothermia after hypoxic–ischemic encephalopathy

**DOI:** 10.3389/fneur.2023.1231743

**Published:** 2023-08-30

**Authors:** Simon C. Dyall, Isabell Nessel, Jennine A. Sharpe, Ping K. Yip, Adina T. Michael-Titus, Divyen K. Shah

**Affiliations:** ^1^School of Life and Health Sciences, University of Roehampton, London, United Kingdom; ^2^Centre for Neuroscience, Surgery and Trauma, Blizard Institute, Barts and The London School of Medicine and Dentistry, Queen Mary University of London, London, United Kingdom; ^3^Royal London Hospital, Barts Health NHS Trust, London, United Kingdom

**Keywords:** DHA, EPA, hypoxic–ischemic encephalopathy, lipid peroxidation, mild therapeutic hypothermia, newborns, Omega-3 Index, omega-3 polyunsaturated fatty acids

## Abstract

Hypoxic–ischemic encephalopathy (HIE) is a major cause of neonatal morbidity and mortality. Although therapeutic hypothermia is an effective treatment, substantial chronic neurological impairment often persists. The long-chain omega-3 polyunsaturated fatty acids (PUFAs), docosahexaenoic (DHA) and eicosapentaenoic (EPA) acids, offer therapeutic potential in the post-acute phase. To understand how PUFAs are affected by HIE and therapeutic hypothermia we quantified for the first time the effects of HIE and therapeutic hypothermia on blood PUFA levels and lipid peroxidation. In a cross-sectional approach, blood samples from newborns with moderate to severe HIE, who underwent therapeutic hypothermia (sHIE group) were compared to samples from newborns with mild HIE, who did not receive therapeutic hypothermia, and controls. The sHIE group was stratified into cerebral MRI predictive of good (*n* = 10), or poor outcomes (*n* = 10; nine developed cerebral palsy). Cell pellets were analyzed for fatty acid content, and plasma for lipid peroxidation products, thiobarbituric acid reactive substances and 4-hydroxy-2-nonenal. Omega-3 Index (% DHA + EPA) was similar between control and HIE groups; however, with therapeutic hypothermia there were significantly lower levels in poor vs. good prognosis sHIE groups. Estimated Δ-6 desaturase activity was significantly lower in sHIE compared to mild HIE and control groups, and linoleic acid significantly increased in the sHIE group with good prognosis. Reduced long-chain omega-3 PUFAs was associated with poor outcome after HIE and therapeutic hypothermia, potentially due to decreased biosynthesis and tissue incorporation. We speculate a potential role for long-chain omega-3 PUFA interventions in addition to existing treatments to improve neurologic outcomes in sHIE.

## 1. Introduction

Hypoxic–ischemic encephalopathy (HIE) is a major cause of neonatal morbidity and mortality worldwide, with an incidence of between 1.0 and 8.0 per 1,000 live births (1). In those who survive there is an increased risk of long-term neurological morbidities, such as cerebral palsy, epilepsy, learning and behavioral problems (2). Mild therapeutic hypothermia is a standard of care in high income countries (3, 4); however, up to 40–50% of infants treated with therapeutic hypothermia die or develop chronic neurological impairment (5). There is therefore an urgent need to develop novel therapeutic strategies that may complement therapeutic hypothermia in the acute phase, but also for interventions in the post-acute repair and regeneration phase that may improve longer term outcomes.

Docosahexaenoic (DHA, 22:6n-3), and arachidonic (ARA, 20:4n-6) acid are long-chain omega-3 and omega-6 polyunsaturated fatty acids (PUFAs), respectively, which are essential for optimal brain and visual system development, and immune system function (6, 7). DHA and other long-chain omega-3 PUFAs, such as eicosapentaenoic acid (EPA, 20:5n-3), are precursors to a wide range of bioactive lipid mediators, such as the specialized proresolving mediators, which exhibit potent neuroprotective and anti-inflammatory properties (8). The initial rate-limiting step in the biosynthesis of the long-chain omega-3 and omega-6 PUFAs is catalyzed by the enzyme, Δ-6 desaturase (D6D) (8).

Omega-3 PUFAs, and particularly DHA, have been widely studied in animal models of hypoxic–ischemic brain injury, where beneficial effects, such as reducing oxidative stress and brain damage, and improving sensorimotor function have been observed (9–12). During the course of therapeutic hypothermia alterations in triacylglycerols and phospholipid species have been identified in newborns with moderate to severe HIE, compared to control samples from newborns with mild HIE (13), suggesting PUFAs may also be altered; however, to the best of our knowledge, the effects of HIE and therapeutic hypothermia on PUFA content have not previously been reported.

During perinatal asphyxia, deprivation of oxygen and glucose initiates a cascade of detrimental processes, such as necrosis, apoptosis, production of reactive oxygen species and subsequent lipid peroxidation, which underlie the pathophysiology of HIE (14). PUFAs are highly susceptible to peroxidation, and the brain is particularly vulnerable to reactive oxygen species-mediated lipid peroxidation due to its high PUFA content (8, 15). Peroxidation of PUFAs produces a range of secondary peroxidation products, such as 4-hydroxy-2-nonenal (4-HNE) and thiobarbituric acid reactive substances (TBARS), including malondialdehyde (MDA) (15, 16). Increased TBARS, MDA, and 4-HNE have consistently been reported following perinatal asphyxia in both umbilical cord blood and serum samples (17, 18).

We measured the effects of therapeutic hypothermia on blood fatty acid profiles in newborns with mild and severe HIE, compared to levels in cord blood from healthy newborns. Blood fatty acid profiles and secondary lipid peroxidation were further investigated over the therapeutic hypothermia phase by measuring TBARS and 4-HNE of newborns with severe HIE, with either favorable or unfavorable brain injury outcome, based on cerebral magnetic resonance imaging (MRI).

## 2. Method

### 2.1. Participants

Between January 2014 and January 2016, term newborns with perinatal asphyxia were recruited after research and ethics approval (REC 13/LO/17380 Bromley, UK) and informed consent from parents, from five tertiary neonatal centers (The Royal London Hospital, Homerton University Hospital, Ashford and St Peter’s Hospitals, Norfolk and Norwich University Hospitals, and Southampton University Hospital) as part of the Brain Injury Biomarkers in Newborns Study (BIBiNS). Infants with moderate to severe HIE were treated with therapeutic hypothermia for 72 h, commencing within 6 h after birth as per standard guidance (3), and as directed by local clinicians. A servo-controlled total body cooling device was used to maintain the core temperature at 33.5°C for 72 h, monitored by rectal probe, and rewarming was carried out over a 12-h period. Additionally, cord blood from healthy newborn babies was collected in the delivery suite at the Royal London Hospital. Exclusion criteria included babies born below 36 weeks gestational age, and babies with multiple congenital abnormalities and/or encephalopathy due to inborn errors of metabolism.

### 2.2. Cerebral MRI outcomes

For this analysis, samples were obtained from 10 healthy control infants (Ctr) (cord blood collected at birth), 10 infants with mild HIE (mHIE) (blood collected within 48 h after birth), and 20 infants with moderate–severe HIE who underwent TH (sHIE), 10 had favorable cerebral MRI (sHIE+) and 10 unfavorable cerebral MRI (sHIE-), as a pragmatic approach. MRI for sHIE infants was performed at local centers with conventional T1-weighted and T2-weighted sequences previously described (19). Images were independently rated by a neuroradiologist and a neonatologist with imaging expertise, blinded to clinical information, wherever disagreements occurred, they were resolved through discussion until a consensus was reached. Patterns of MRI injury were classified into two groups derived from the system described by Rutherford and co-workers (20), which has prognostic value in infants who have undergone therapeutic hypothermia. Infants with an unfavorable outcome had a severe pattern of injury including reversed or abnormal signal intensity bilaterally on T1- and/or T2-weighted sequences in the posterior limb of the internal capsule (PLIC); multifocal or widespread abnormal signal intensity in the basal ganglia and thalami (BGT); and severe widespread white matter (WM) lesions including infarction, hemorrhage and long T1 and T2. Infants with MRIs predictive of a favorable outcome had either normal images or less severe patterns of injury that are associated with normal or only mildly abnormal neurodevelopmental outcomes. In this cohort we have shown that using this method, cerebral MRI is highly predictive of neurodevelopmental outcomes (19).

### 2.3. Neurodevelopmental assessments

Neurodevelopmental assessments for infants with sHIE were carried out using Bayley III assessments as part of local clinical care (21). The diagnosis of cerebral palsy was assigned by local clinical teams. For children with cerebral palsy where cognitive disability was too severe to allow testing, a score of 55 (−3 standard deviations (SD)) was assigned.

### 2.4. Blood samples

Blood samples were taken from an arterial line where available, or from venipuncture, or heel prick, when sampling was carried out for routine clinical care. In sHIE groups, blood was taken at three time points throughout the therapeutic hypothermia phase: sample 1 (S1) after reaching target temperature (12–24 h after birth); sample 2 (S2) prior to rewarming (approximately 48 h after birth) and sample 3 (S3) after rewarming (around 96 h after birth). Blood was collected in ethylenediaminetetraacetic acid tubes, and centrifuged at 1000 × *g* for 10 min at room temperature within 3 h, before plasma was separated from cell pellet and both were stored at −20°C for maximum 3 months, and long-time storage at −70°C. Samples were not available for all infants at all time-points, and the sample availability for all outcome measures is reported in Supplementary Table S1. Information on parenteral and enteral nutritional intake was collected for participants in the sHIE groups undergoing therapeutic hypothermia, including if/when feeds commenced and the type of feed (e.g., expressed breast milk, donor milk, or formula milk), and is shown in Supplementary Table S2.

### 2.5. Fatty acid profile and desaturase activity

Lipids were extracted from 100 μL of cell pellet using a modified Bligh and Dyer method described by Bell and colleagues (22), using methanol and chloroform. Heptadecanoic acid (25 mg/100 mL methanol, Sigma) was added as internal standard. The oily residue was transesterified using BF_3_-methanol (23), and samples were analyzed by gas chromatography with flame ionization detection (7820A; Agilent Technologies, Santa Clara, CA, USA), using an Omegawax^™^ column (30 m × 0.25 mm × 0.25 μm, Supelco). Fatty acid methyl ester identity was confirmed compared to retention times of analytical standards, and values corrected for detector response. The Omega-3 Index was calculated as the sum of EPA and DHA as percentage of total fatty acids (24). Desaturase activities were estimated based on product-to-precursor ratios: D6D activity = 18:3n-6/18:2n-6, and Δ-5 desaturase (D5D) activity = 20:4n-6/20:3n-6 (25).

### 2.6. Quantification of TBARS

TBARS, including MDA, in plasma were measured using a commercial assay (TBARS TCA method, Cayman Chemical), following manufacturer’s instructions. Samples were incubated with trichloric acid and color reagent and absorbance of duplicates measured at 540 nm.

### 2.7. Quantification of 4-HNE

4-HNE adducts were quantified using an OXISelect HNE adduct competitive ELISA assay (Cell Biolabs), according to manufacturer’s instructions. Plates were coated overnight, and samples were incubated before addition of anti-HNE antibody. Absorbance was measured at 450 nm following incubation with secondary antibody. 11 samples (1 control, 1 mHIE, 4 sHIE+ (2 subjects), and 5 sHIE- (4 subjects)) were below the limit of quantification (1.56 μg/mL), and values were set at the lower level of quantification, to avoid potential bias in the results (26).

### 2.8. Statistical analysis

The strengthening the reporting of observational studies in epidemiology (STROBE) guidelines were followed (27). Statistical analysis was performed using GraphPad Prism (Version 9.3.1). Data were tested for normality using the Shapiro–Wilk test. Participant characteristics and Ctr, mHIE and sHIE S1 levels were compared using Chi square, one-way ANOVA or Kruskal-Wallis tests, with *post hoc* analysis undertaken using Tukey’s or Dunn tests, respectively. Time course analysis was performed using two-way ANOVA, with Bonferroni’s test applied where appropriate. Data are presented as mean ± SD, median [IQR], or median [25% percentile, 75% percentile], and considered statistically significant at *p* < 0.05. The detailed abbreviations and definitions used in the paper are listed in Supplementary Table S3.

## 3. Results

### 3.1. Participants and samples

The perinatal characteristics of the 40 newborns included in this study are shown in Table 1. Babies in the sHIE group had lower APGAR scores, were more likely to be treated with respiratory support, inotropes and additional blood products. The nutritional intake of the sHIE groups is summarized in Supplementary Table S2.

**Table 1:** Clinical characteristics of participants.

	Controls	Mild HIE	sHIE+	sHIE-	*P*-value
*N*	10	10	10	10	
Male, n (%)	7 (70%)	1 (10%)	3 (30%)	10 (100%)	<0.001^1^
Birth weight (Kg)	3.17 [2.79, 3.43]	3.33 [3.05, 3.73]	3.79 [3.32, 4.27]	3.47 [3.00, 3.81]	NS^2^
APGAR at 10 min	10 [10, 10]	10 [8, 10]	3 [1, 5] ^# $^	4 [4, 9] ^# $^	<0.001^2^
Chest compression, n (%)	0 (0%)	1 (10%)	4 (40%)	3 (30%)	NS^1^
Worst pH within 1 h	N/A	7.0 [6.9, 6.9]	6.9 [6.8, 7.0]	6.9 [6.8, 7.0] (*n* = 9)	NS^2^
Worst base deficit < 1 h	−6.4 [−6.4, −6.4] (*n* = 1)	−10.7 [−13.9, −9.6]	−18.1 [−21.34, −15.7] ^$^	−17.3 [−19.7, −15.3] ^$^ (*n* = 8)	0.006^2^
Worst lactate < 1 h	N/A	11.0 [9.5, 13.8]	9.6 [8.2, 12.4] (*n* = 8)	18.0 [14.2, 21.0] ^$Ŧ^ (*n* = 7)	0.008^2^
Hypotension, n (%)	0 (0%)	0 (0%)	4 (40%)	7 (70%)	<0.001^1^
Blood products, n (%)	0 (0%)	0 (0%)	3 (30%) 3x PRBC, 1x FFP/platelets	6 (67%) (*n* = 9) 3x PRBC, 3x platelets, 3x FFP	0.001^1^
Sentinel event, n (%)	0 (0%)	1 (10%)	4 (40%) 3x Sd, 1x PA	2 (20%) 2x Sd	NS^1^
Alive at discharge, n (%)	10 (100%)	10 (100%)	10 (100%)	9 (90%)	NS^1^

FFP, Fresh Frozen Plasma; NS, not significant; PA, placental abruption; PRBC, Packed Red Blood cells; Sd, shoulder dislocation. If dataset is incomplete, n numbers have been provided in the table, data are presented as median [25% percentile, 75% percentile] or n (%). ^1^ Groups were compared by Chi square test and ^2^ groups were compared by Kruskal-Wallis. ^#^ Compared to control, ^$^ compared to mHIE and ^Ŧ^ compared to sHIE+.

### 3.2. Cerebral MRI and neurodevelopmental outcomes

In the 10 babies with cerebral MR images predictive of a good outcome (sHIE+), six had completely normal images, one had moderate WM changes, two had mild BGT changes and one had mild WM and BGT changes. Of the 10 babies with images predictive of poor outcomes (sHIE-), five had abnormalities in all three domains of PLIC, BGT and WM, three had abnormalities in PLIC and BGT, and two had abnormalities in BGT and WM.

The Bayley composite scores were 120 [41] for cognition, 110 [17] for language and 105 [12] for motor outcomes for babies in the sHIE+ group. Testing in this group was carried out at a median age of 2.2 years. In the sHIE- group, nine of the 10 children were classified by the local team as having severe cerebral palsy and were assigned scores of 55 for all domains. The other child was assigned scores of 55 in the cognition and language domains, but had a motor score of 100.

### 3.3. Fatty acids

**Figure 1:**
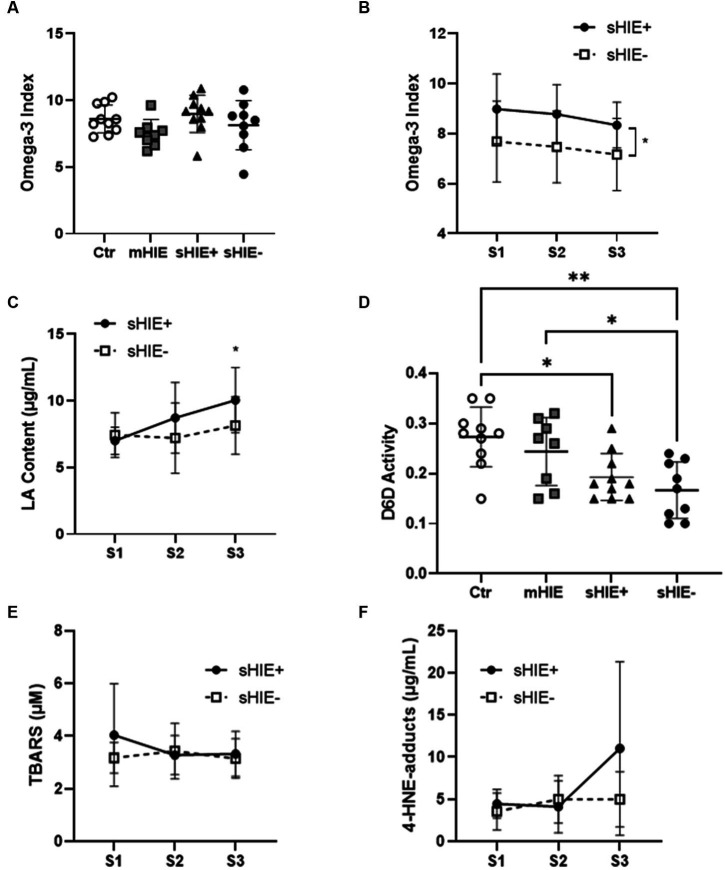
Selected polyunsaturated fatty acid and lipid peroxidation analysis results. **(A)** Omega-3 Index of Ctr, mHIE, S1 sHIE+ and S1 sHIE- were not significantly different. **(B)** Omega-3 Index over the therapeutic hypothermia phase, were there were significant differences between groups (*p* < 0.05). **(C)** LA content over the therapeutic hypothermia phase, where there were significant differences in sHIE+ between S1 and S3 (*p* < 0.05). **(D)** Estimated Δ-6 desaturase activity of control, mHIE and S1 sHIE+ and S1 sHIE-, where there were significant differences between the groups. **(E)** Thiobarbituric acid reactive substances levels did not significantly change over the therapeutic hypothermia phase. **(F)** 4-Hydroxy-2-nonenal-adduct levels did not significantly change over the therapeutic hypothermia phase. **(A,D)** were analyzed by one-way ANOVA, whereas **(B,C,E,F)** were analyzed by two-way ANOVA, * represents *p* < 0.05 and **p < 0.01.

Relative fatty acid levels are shown in Table 2, and absolute values in Table 3. There were no significant differences in absolute or relative content of DHA, EPA, or Omega-3 Index between the Ctr, mHIE, and S1 sHIE groups (Figure 1A). Similarly, there was no significant change over time in absolute or relative EPA and DHA levels in the sHIE samples. However, there was a significant difference in Omega-3 Index between infants in the sHIE+ and sHIE- group over the therapeutic hypothermia phase (S1, S2, and S3) (*F*(1, 17) = 6.14, *p* = 0.04), with the sHIE+ groups having approximately 10% higher levels (*p* = 0.04, Figure 1B). Although there were trends for decreases in the Omega-3 Index levels of both groups over the therapeutic hypothermia phase, these were not statistically significant.

**Table 2:** Erythrocyte fatty acid composition (% total fatty acids).

FA	Ctr	mHIE	sHIE+ S1	sHIE+ S2	sHIE+ S3	sHIE- S1	sHIE- S2	sHIE- S3
16:0	28.14 ± 1.07	28.34 ± 1.21	27.53 ± 1.29	27.40 ± 1.22	27.81 ± 1.08	29.00 ± 2.46	30.63 ± 4.13	29.77 ± 2.92
18:0	19.31 ± 0.78	19.06 ± 2.26	18.04 ± 1.40	17.38 ± 1.64	15.99 ± 0.94	17.96 ± 1.83	18.38 ± 2.88	18.81 ± 5.47
20:0	0.49 ± 0.08	0.54 ± 0.12	0.48 ± 0.06	0.43 ± 0.06	0.45 ± 0.10	0.45 ± 0.07	0.55 ± 0.14	0.43 ± 0.09
22:0	0.40 ± 0.16	0.46 ± 0.15	0.51 ± 0.21	0.45 ± 0.23	0.49 ± 0.16	0.46 ± 0.16	0.42 ± 0.13	0.39±0.17
24:0	1.81 ± 0.85	1.37 ± 1.36	1.30 ± 0.17	1.25 ± 0.28	1.10 ± 0.28	1.59 ± 0.46	1.37 ± 0.59	1.34 ± 0.65
16:1n-7	0.61 ± 0.29	0.52 ± 0.30	0.61 ± 0.29	0.53 ± 0.23	0.66 ± 0.29	0.61 ± 0.25	0.55 ± 0.19	0.74 ± 0.25
18:1n-9	9.53 ± 0.60	12.00 ± 0.74	12.20 ± 1.55	12.97 ± 1.73	14.15 ± 1.91	11.74 ± 2.23	12.50 ± 1.68	12.77 ± 1.92
18:1n-7	1.88 ± 0.25	2.14 ± 0.44	2.03 ± 0.60	2.09 ± 0.44	1.92 ± 0.31	1.96 ± 0.22	2.19 ± 0.58	1.95 ± 0.38
20:1n-9	0.19 ± 0.09	0.26 ± 0.06	0.20 ± 0.06	0.24 ± 0.06	0.23 ± 0.04	0.23 ± 0.10	0.27 ± 0.17	0.24 ± 0.15
22:1n-9	0.15 ± 0.06	0.14 ± 0.03	0.21 ± 0.11	0.15 ± 0.05	0.14 ± 0.04	0.16 ± 0.06	0.18 ± 0.06	0.13 ± 0.04
24:1n-9	0.82 ± 0.22	0.83 ± 0.28	1.15 ± 0.37	0.91 ± 0.27	0.77 ± 0.14	0.98 ± 0.25	1.31 ± 0.46	0.90 ± 0.18
18:2n-6 (LA)	3.80 ± 0.43	4.11 ± 0.64	4.46 ± 0.60	5.24 ± 0.90	5.96 ± 1.59	4.69 ± 1.21	4.84 ± 1.56	5.07 ± 1.12
18:3n-6	1.02 ± 0.20	1.01 ± 0.34	0.86 ± 0.27	1.03 ± 0.57	0.89 ± 0.26	0.79 ± 0.33	0.85 ± 0.32	0.85 ± 0.41
20:2n-6	0.32 ± 0.15	0.55 ± 0.25	0.51 ± 0.21	0.45 ± 0.18	0.49 ± 0.16	0.59 ± 0.41	0.45 ± 0.23	0.37 ± 0.16
20:3n-6	2.74 ± 0.35	2.89 ± 0.34	2.78 ± 0.25	2.59 ± 0.43	2.46 ± 0.36	2.44 ± 0.55	2.10 ± 0.70	2.07 ± 0.82
20:4n-6 (ARA)	17.74 ± 1.09	16.35 ± 1.48	16.22 ± 1.31	16.16 ± 1.58	16.35 ± 1.62	16.16 ± 2.11	14.18 ± 4.54	15.19 ± 5.47
22:4n-6	4.19 ± 0.65	3.53 ± 0.58	3.66 ± 0.40	3.48 ± 0.48	3.17 ± 0.60	3.85 ± 0.60	3.15 ± 1.03	3.15 ± 1.18
18:3n-3	0.30 ± 0.07	0.16 ± 0.04	0.20 ± 0.08	0.21 ± 0.09	0.22 ± 0.08	0.19 ± 0.08	0.20 ± 0.08	0.18 ± 0.07
18:4n-3	0.13 ± 0.16	0.05 ± 0.01	0.06 ± 0.01	0.07 ± 0.02	0.06 ± 0.02	0.06 ± 0.02	0.05 ± 0.02	0.06 ± 0.02
20:3n-3	0.13 ± 0.04	0.09 ± 0.04	0.09 ± 0.02	0.10 ± 0.05	0.07 ± 0.03	0.08 ± 0.04	0.09 ± 0.03	0.08 ± 0.02
20:5n-3 (EPA)	0.78 ± 0.37	0.56 ± 0.11	0.61 ± 0.11	0.59 ± 0.16	0.51 ± 0.06	0.63 ± 0.16	0.67 ± 0.19	0.62 ± 0.18
22:5n-3	0.60 ± 0.28	0.70 ± 0.38	0.80 ± 0.22	0.90 ± 0.36	0.98 ± 0.35	0.65 ± 0.35	0.48 ± 0.37	0.63 ± 0.44
22:6n-3 (DHA)	7.83 ± 0.84	6.96 ± 0.94	8.38 ± 1.39	8.19 ± 1.16	7.83 ± 0.84	7.50 ± 1.79	7.14 ± 1.62	6.76 ± 1.43

ARA, arachidonic acid; DHA, docosahexaenoic acid; EPA, eicosapentaenoic acid; LA, linoleic acid. Data are presented as mean ± standard deviation.

**Table 3:** Erythrocyte fatty acid composition (μg/mL).

FA	Ctr	mHIE	sHIE+ S1	sHIE+ S2	sHIE+ S3	sHIE- S1	sHIE- S2	sHIE- S3
16:0	44.86 ± 4.1.4	49.55 ± 6.71	43.25 ± 3.23	44.72 ± 4.76	47.31 ± 4.43	46.51 ± 5.46	45.15 ± 5.05	48.02 ± 8.38
18:0	30.84 ± 3.52	33.88 ± 8.82	28.49 ± 4.09	28.70 ± 6.26	27.23 ± 3.10	28.97 ± 4.74	27.14 ± 4.10	30.15 ± 8.37
20:0	0.79 ± 0.14	0.96 ± 0.36	0.75 ± 0.14	0.70 ± 0.14	0.75 ± 0.14	0.72 ± 0.015	0.81 ± 0.19	0.68 ± 0.10
22:0	0.63 ± 0.22	0.78 ± 0.21	0.79 ± 0.38	0.69 ± 0.34	0.82 ± 0.29	0.74 ± 0.25	0.63 ± 0.22	0.63 ± 0.29
24:0	2.85 ± 1.21	2.30 ± 0.30	1.93 ± 0.31	1.89 ± 0.38	1.79 ± 0.56	2.60 ± 0.92	2.17 ± 1.09	2.29 ± 1.23
16:1n-7	0.97 ± 0.47	0.88 ± 0.45	0.96 ± 0.50	0.85 ± 0.36	1.11 ± 0.43	0.96 ± 0.36	0.81 ± 0.23	1.17 ± 0.40
18:1n-9	19.21 ± 1.79	21.04 ± 3.29	19.23 ± 3.00	21.34 ± 4.44	20.23 ± 4.85	18.76 ± 3.32	18.56 ± 3.17	20.45 ± 3.52
18:1n-7	3.00 ± 0.47	3.71 ± 0.73	3.21 ± 1.08	3.50 ± 1.26	3.27 ± 0.57	3.14 ± 0.45	3.32 ± 1.22	3.10 ± 0.52
20:1n-9	0.31 ± 0.14	0.37 ± 0.14	0.33 ± 0.11	0.40 ± 0.14	0.39 ± 0.09	0.37 ± 0.14	0.38 ± 0.19	0.34 ± 0.13
22:1n-9	0.24 ± 0.09	0.24 ± 0.05	0.31 ± 0.17	0.22 ± 0.06	0.22 ± 0.06	0.26 ± 0.10	0.27 ± 0.12	0.21 ± 0.07
24:1n-9	1.31 ± 0.34	1.39 ± 0.54	1.63 ± 0.51	1.35 ± 0.44	1.19 ± 0.27	1.59 ± 0.52	1.97 ± 0.84	1.44 ± 0.32
18:2n-6 (LA)	6.09 ± 1.12	7.24 ± 1.58	7.00 ± 0.96	8.72 ± 2.51	10.05 ± 2.32	7.34 ± 1.57	7.20 ± 0.09	8.15 ± 2.03
18:3n-6	1.61 ± 0.24	1.72 ± 0.50	1.35 ± 0.39	1.80 ± 1.30	1.52 ± 0.51	1.25 ± 0.48	1.23 ± 0.32	1.30 ± 0.43
20:2n-6	0.52 ± 0.25	0.95 ± 0.43	0.82 ± 0.36	0.73 ± 0.31	0.85 ± 0.30	0.94 ± 0.60	0.71 ± 0.43	0.62 ± 0.31
20:3n-6	4.36 ± 0.63	5.05 ± 0.89	4.37 ± 0.47	4.17 ± 0.51	4.21 ± 0.89	3.92 ± 0.92	3.21 ± 1.26	3.43 ± 1.51
20:4n-6 (ARA)	28.29 ± 3.21	28.28 ± 5.13	24.69 ± 2.19	25.46 ± 2.45	27.10 ± 4.29	26.08 ± 4.73	22.07 ± 8.52	25.49 ± 10.41
22:4n-6	6.71 ± 1.33	5.92 ± 0.61	5.38 ± 0.46	5.28 ± 0.48	5.08 ± 1.14	6.21 ± 1.20	4.91 ± 1.94	5.28 ± 2.22
18:3n-3	0.47 ± 0.11	0.28 ± 0.07	0.31 ± 0.13	0.33 ± 0.12	0.37 ± 0.12	0.31 ± 0.12	0.30 ± 0.09	0.27 ± 0.06
18:4n-3	0.21 ± 0.26	0.09 ± 0.02	0.09 ± 0.02	0.11 ± 0.04	0.10 ± 0.03	0.10 ± 0.03	0.08 ± 0.02	0.10 ± 0.03
20:3n-3	0.21 ± 0.07	0.16 ± 0.07	0.13 ± 0.04	0.15 ± 0.07	0.12 ± 0.04	0.12 ± 0.05	0.13 ± 0.05	0.13 ± 0.05
20:5n-3 (EPA)	1.23 ± 0.58	0.95 ± 0.18	0.93 ± 0.21	0.91 ± 0.22	0.84 ± 0.08	1.02 ± 0.29	0.97 ± 0.24	0.98 ± 0.24
22:5n-3	0.96 ± 0.49	1.19 ± 0.27	1.18 ± 0.29	1.41 ± 0.65	1.54 ± 0.45	1.04 ± 0.54	0.74 ± 0.57	1.03 ± 0.72
22:6n-3 (DHA)	12.55 ± 2.34	11.63 ± 1.70	11.84 ± 1.56	12.11 ± 1.98	12.10 ± 1.79	12.08 ± 3.17	10.90 ± 3.32	11.17 ± 3.17

ARA, arachidonic acid; DHA, docosahexaenoic acid; EPA, eicosapentaenoic acid; LA, linoleic acid. Data are presented as mean ± standard deviation.

There were no significant differences in absolute and relative levels of the omega-6 PUFAs, linoleic acid (LA, 18:6n-6), ARA and adrenic acid (22:4n-6) between the Ctr, mHIE, and sHIE groups. Similarly, there was no significant change in absolute or relative ARA or adrenic acid levels over the therapeutic hypothermia phase. However, there was a significant increase in the absolute levels of LA over the time of therapeutic hypothermia (*F*(2, 51) = 3.62, *p* = 0.03), with significantly higher levels in S3, compared to S1 in the sHIE+ group (*p* = 0.03, Figure 1C). Supplementary Table S2 summarizes the feeding data for the sHIE groups over the therapeutic hypothermia period, where it can be seen that 20% (2/10) of the sHIE+ group were nil-by-mouth, whereas this was 44% (4/9) for the sHIE- group. Furthermore, the feeds generally commenced earlier in the sHIE+ group.

### 3.4. Estimated desaturase activity

The estimated D6D activity was significantly different between the Ctr and HIE groups (*F*(93, 33) = 6.606, *p* = 0.001) (Figure 1D), with significantly lower activity in sHIE+ (*p* = 0.019) and sHIE- (*p* = 0.002) groups compared to the Ctr group, and significantly lower activity in sHIE- compared to mHIE (*p* = 0.044). No change in D6D activity was seen over the therapeutic hypothermia phase. There was no significant difference in mean ± SD D5D activity between the groups, Ctr 6.57 ± 0.77 vs. mHIE 5.74 ± 0.85 vs. sHIE+ 5.89 ± 0.77 vs. sHIE- 6.91 ± 1.60, or over the therapeutic hypothermia phase.

### 3.5. TBARS

S1 mean ± SD TBARS levels for sHIE+ and sHIE- groups were, 4.04 ± 1.94 μM, and 3.17 ± 0.58 μM, respectively. No significant changes in TBARS levels were seen in either group over the therapeutic hypothermia phase, Figure 1E.

### 3.6. 4-HNE

S1 mean ± SD 4-HNE-adduct levels for sHIE+ and sHIE- groups were 4.45 ± 1.70 μg/mL, and 3.56 ± 2.19 μg/mL, respectively. No significant changes in 4-HNE-adduct levels were seen in either group over the therapeutic hypothermia phase, Figure 1F.

## 4. Discussion

This exploratory study measured for the first time the effects of therapeutic hypothermia and HIE on fatty acid profiles and the lipid peroxidation products, TBARS and 4-HNE, in neonates. Neonates with substantial brain injury on MRI after HIE and therapeutic hypothermia, nine out 10 of whom went on to develop cerebral palsy, had a significantly lower Omega-3 Index. A significantly lower level of estimated D6D activity was identified in infants with sHIE than in controls or mHIE. Although D6D activity remained constant over the therapeutic hypothermia and rewarming period, there was a significant increase in LA in the severe group, potentially indicating low conversion to longer chain PUFAs. There were no overall significant differences in ARA, DHA or EPA content between the groups, and levels were maintained over the therapeutic hypothermia phase. There were no significant differences in TBARS or 4-HNE content between the sHIE groups, or over the therapeutic hypothermia phase.

No significant differences in ARA, DHA or EPA were identified between the groups. Comparisons with published values when the contents are expressed as percent composition are complicated, due to the results being strongly interdependent and varying depending on the number of fatty acids analyzed and presented. As there is not a standardized list of fatty acids to be included in the total fatty acids, differences in the number of summed fatty acids in the analyses will affect the apparent values of the reported fatty acids (28, 29). Nonetheless, the values reported in this study are consistent with others (30, 31), suggesting that none of the groups were deficient. Furthermore, the mean Omega-3 Index levels for all groups were above values of 6%, considered beneficial in children (32). However, when expressed in absolute levels, the levels of all fatty acids were below those reported by others (30), although it should be noted that this analysis was based on whole blood from a dried blood spot analysis, which is a global measure of total circulating fatty acids, including both plasma and all blood cells, whereas in the present study only the cell pellet from whole blood was analyzed. It should also be acknowledged that this pellet may also contain leukocytes, which differ in composition to erythrocytes (33).

There were significant differences in the Omega-3 Index between the sHIE groups, with higher levels seen in the good vs. poor MRI outcome groups. The Omega-3 Index has been shown to correlate with DHA and EPA content of other cells, and is therefore considered a good indicator of DHA and EPA status (24, 34), and may therefore be more sensitive than measuring DHA or EPA separately. These observations, suggesting that a lower Omega-3 Index was associated with a poorer prognosis, are consistent with other studies showing that a higher omega-3 PUFA content confers neuroprotection against neonatal HI brain injury (35).

The ARA, DHA, and EPA levels did not significantly change over the period of therapeutic hypothermia and rewarming, and although there were trends for decreases in Omega-3 Index, this was not significant, suggesting that ARA, DHA and EPA levels were somewhat maintained during therapeutic hypothermia and rewarming. However, in both groups undergoing therapeutic hypothermia, there were increases in LA, which were significant in the sHIE+ group. Due to the small sample size of these groups, it was not possible to perform statistical analysis on the effects of nutritional intake. However, in the sHIE+ group six received breast milk, two received formula milk and only two were nil-by-mouth, whereas in the sHIE- group four were nil-by-mouth, three received expressed breast milk, one donor human milk, and none received formula milk. The observed increases in LA levels may therefore be a consequence of this nutritional intake, as formula milk, expressed breast milk and donor human milk are dietary sources high in LA, and low in DHA and ARA (36, 37). High LA intake decreases DHA biosynthesis and the incorporation of DHA into tissues (38).

Endogenous biosynthesis of long-chain omega-3 and omega-6 PUFAs is regulated by D6D, as it is a rate-limiting enzyme in their pathways (8). D6D activity is associated with metabolic risks, including type 2 diabetes (39–41). There is a low placental transfer of LA (42), which may be a specific biological mechanism to enable higher rates of Δ-6 desaturation of omega-3 and omega-6 PUFAs (43). At birth, both term and preterm infants have higher D6D activity than mothers (43, 44). D6D activity was shown to decline to about one-third at 1 month, then further decreases to about one-sixth at 6 months, when it remained stable until 12 months (44). In comparison to the values reported in these studies, low D6D activity was observed in all groups in the present study, suggesting particularly low rates of long-chain PUFA biosynthesis in the sHIE groups.

The results suggest a potential role for additional EPA and DHA in addition to existing treatments; however, there is currently somewhat equivocal evidence from experimental animal models of HIE, as to the potential for DHA to act as an effective adjuvant treatment and enhance the therapeutic effects of therapeutic hypothermia in hypoxic ischemic brain injury. A study in a neonatal rat model of hypoxic ischemic injury reported that therapeutic hypothermia plus DHA synergistically reduced brain infarct volume and improved behavioral performance, as assessed 1 week after injury (45). A study in newborn piglets (within 36 h after birth) showed that combining DHA with therapeutic hypothermia does not appear to provide additional benefits to either therapeutic hypothermia or DHA alone, when the hypoxic ischemic injury outcomes were assessed at 9.5 h after injury (12). In a more recent study in neonatal mice (10-day old) subjected to hypoxic ischemic injury, DHA and therapeutic hypothermia did not synergize, as assessed in terms of infarct size at 24 h post-injury (12, 46). Therefore, these reports in different species and with assessment of different outcomes at varied times post-injury, do not allow to reach a definitive conclusion as to the additional potential benefit of using DHA in therapeutic hypothermia-exposed neonates. However, the low D6D activity observed in the sHIE groups suggests an increased requirement for preformed ARA and DHA, which should be coupled concomitantly with a decreased LA intake, to compensate for the potentially lower endogenous synthesis of the long-chain PUFAs and decreased incorporation into tissues. Furthermore, positive results from the Dolphin neonatal trial show the feasibility of DHA-based interventions in this clinical population in the post-acute phase (47).

The levels of TBARS and 4-HNE remained constant over the course of therapeutic hypothermia and rewarming, suggesting that the procedure prevented any injury-induced increases in lipid peroxidation. Mild hypothermia following traumatic brain injury has been shown to enhance the activity of the antioxidant enzymes superoxide dismutase and glutathione peroxidase and reduce MDA in mice (48), and following severe global hypoxia in piglets, therapeutic hypothermia reduced lipid peroxidation in white matter, although not in cortical gray matter (49). Furthermore, compared to normothermia treatment, therapeutic hypothermia significantly increased blood total antioxidant score, and significantly decreased MDA and the risk of developing neurological deficit in a randomized controlled trial of neonates with perinatal asphyxia (50).

This was a pragmatic exploratory pilot study in which blood samples obtained from four different groups were compared; within the moderate to severe HIE group comparison was made between 10 babies with very good outcomes and nine babies with extremely poor outcomes. As such this is not a cohort study. Our study used product/precursor ratios to estimate desaturase activities; however, traditionally activities are quantified using radio-labeled substrates and measuring the rate of appearance of the radioactive product. This approach is gaining greater acceptance in the literature, and the two techniques correlate well, although the strength of the relationship appears dependent on the lipid fraction analyzed for fatty acid composition (51). However, as this is not a direct measure of enzyme activity, the results should be interpreted with care. Moreover, further work should explore other aspects of PUFA metabolism, such as oxylipin production and also the activities of the enzymes responsible for their hydrolysis, such as soluble and microsomal epoxide hydrolases (52). Only TBARS and 4-HNE were assessed for lipid peroxidation, and the inclusion of a complementary analysis of non-enzymatically oxidized PUFA products, such as isoprostanes and neuroprostanes, would provide additional information on lipid peroxidation of specific PUFA species (8). Due to ethical and logistical constraints it was not possible to compare the Ctr, mHIE and sHIE groups under the exact same conditions and timepoint, and therefore a pragmatic approach was taken to establish these group comparisons. A range of blood products were administered to the sHIE groups, of which three in the sHIE+ and two in the sHIE- group received packed red blood cells, which may constitute a source of long-chain PUFAs not considered in the analysis. Due to the preliminary nature of this exploratory study and small sample size it was not possible to consider these confounding factors, and others such as, participant’s sex, mothers’ age at delivery, mode of delivery, PUFAs supplied through parenteral and/or enteral nutrition, and type of feedings in our analysis. Future work should seek to explore these in greater detail. It must also be acknowledged that a clinical study such as ours is unable to separate the effect of HIE from the effect of therapeutic hypothermia.

In conclusion, the results indicate that newborns with moderate to severe HIE undergoing therapeutic hypothermia have decreased D6D activity, and increased LA levels, which may lead to decreases in tissue levels of LC-PUFAs. Furthermore, a higher Omega-3 Index was associated with good outcomes as compared to newborns with adverse outcomes, nine out of 10 of whom went on to develop cerebral palsy. Future studies are needed to assess the longer-term effects of HIE and therapeutic hypothermia on infant fatty acid status and lipid peroxidation, in order to improve understanding of the potential role of omega-3 PUFA supplementation in developing more effective neuroprotective and regenerative strategies for perinatal HIE, and to ultimately prevent cerebral palsy.

## Data availability statement

The raw data supporting the conclusions of this article will be made available by the authors, without undue reservation.

## Ethics statement

The studies involving humans were approved by National Research Ethics Service Committee London – Bromley, REC 13/LO/17380 Bromley, UK. The studies were conducted in accordance with the local legislation and institutional requirements. Written informed consent for participation in this study was provided by the participants’ legal guardians/next of kin.

## Author contributions

SD, IN, and DS: conceptualization, methodology, data curation, and writing—original draft preparation. SD, IN, JS, and DS: formal analysis and investigation. SD, IN, PY, AM-T, and DS: resources and writing—review and editing. SD, IN, PY, and DS: project administration. SD, PY, AM-T, and DS: funding acquisition. All authors contributed to the article and approved the submitted version.

## Funding

This study was facilitated by a grant from the Barts Charity (498/1747).

## Conflict of interest

The authors declare that the research was conducted in the absence of any commercial or financial relationships that could be construed as a potential conflict of interest.

## Publisher’s note

All claims expressed in this article are solely those of the authors and do not necessarily represent those of their affiliated organizations, or those of the publisher, the editors and the reviewers. Any product that may be evaluated in this article, or claim that may be made by its manufacturer, is not guaranteed or endorsed by the publisher.

## Abbreviations

4-HNE: 4-hydroxy-2-nonenal
ARA: arachidonic acid
BGT: basal ganglia and thalami
Ctr: control
D5D: Δ-5 desaturase
D6D: Δ-6 desaturase
DHA: docosahexaenoic acid
EPA: eicosapentaenoic acid
HIE: hypoxic ischemic encephalopathy
LA: linoleic acid
MDA: malondialdehyde
mHIE: mild HIE
PLIC: posterior limb of the internal capsule
PUFA: polyunsaturated fatty acid
sHIE: severe HIE
TBARS: thiobarbituric acid reactive substances
WM: white matter

## References

[1] Kurinczuk JJ White-Koning M Badawi N. Epidemiology of neonatal encephalopathy and hypoxic-ischaemic encephalopathy. Early Hum Dev. (2010) 86:329–38. doi: 10.1016/j.earlhumdev.2010.05.010 20554402

[2] Allen KA Brandon DH. Hypoxic ischemic encephalopathy: pathophysiology and experimental treatments. Newborn Infant Nurs Rev. (2011) 11:125–33. doi: 10.1053/j.nainr.2011.07.004, PMID: 21927583 PMC3171747

[3] NICE. Therapeutic hypothermia with intracorporeal temperature monitoring for hypoxic perinatal brain injury. Manchester: NICE (2010).

[4] Committee on Fetus and Newborn Newborn LAP Baley JE Benitz W Cummings J Carlo WA. Hypothermia and neonatal encephalopathy. Pediatrics. (2014) 133:1146–50. doi: 10.1542/peds.2014-0899 24864176

[5] Higgins RD Shankaran S. Hypothermia: novel approaches for premature infants. Early Hum Dev. (2011) 87 Suppl 1:S17–8. doi: 10.1016/j.earlhumdev.2011.01.004, PMID: 21277717 PMC3058821

[6] Dyall SC. Long-chain omega-3 fatty acids and the brain: a review of the independent and shared effects of EPA, DPA and DHA. Front. Aging Neurosci. (2015) 7:52. doi: 10.3389/fnagi.2015.00052, PMID: 25954194 PMC4404917

[7] Tallima H El Ridi R. Arachidonic acid: physiological roles and potential health benefits - a review. J Adv Res. (2018) 11:33–41. doi: 10.1016/j.jare.2017.11.004, PMID: 30034874 PMC6052655

[8] Dyall SC Balas L Bazan NG Brenna JT Chiang N da Costa Souza F. Polyunsaturated fatty acids and fatty acid-derived lipid mediators: recent advances in the understanding of their biosynthesis, structures, and functions. Prog Lipid Res. (2022) 86:101165. doi: 10.1016/j.plipres.2022.101165, PMID: 35508275 PMC9346631

[9] Berman DR Liu Y Barks J Mozurkewich E. Treatment with docosahexaenoic acid after hypoxia-ischemia improves forepaw placing in a rat model of perinatal hypoxia-ischemia. Am J Obstet Gynecol. (2010) 203:385.e1. doi: 10.1016/j.ajog.2010.06.017, PMID: 20691409 PMC2947568

[10] Williams JJ Mayurasakorn K Vannucci SJ Mastropietro C Bazan NG Ten VS. N-3 fatty acid rich triglyceride emulsions are neuroprotective after cerebral hypoxic-ischemic injury in neonatal mice. PLoS One. (2013) 8:e56233. doi: 10.1371/journal.pone.0056233, PMID: 23437099 PMC3577805

[11] Huun MU Garberg HT Escobar J Chafer C Vento M Holme IM. DHA reduces oxidative stress following hypoxia-ischemia in newborn piglets: a study of lipid peroxidation products in urine and plasma. J Perinat Med. (2018) 46:209–17. doi: 10.1515/jpm-2016-0334, PMID: 28632497

[12] Huun MU Garberg H Loberg EM Escobar J Martinez-Orgado J Saugstad OD. DHA and therapeutic hypothermia in a short-term follow-up piglet model of hypoxia-ischemia: effects on H+MRS biomarkers. PLoS One. (2018) 13:e0201895. doi: 10.1371/journal.pone.0201895, PMID: 30086156 PMC6080779

[13] Nixon R Ip THR Jenkins B Yip PK Clarke P Ponnusamy V. Lipid profiles from dried blood spots reveal lipidomic signatures of newborns undergoing mild therapeutic hypothermia after hypoxic-ischemic encephalopathy. Nutrients. (2021) 13:4301. doi: 10.3390/nu13124301, PMID: 34959853 PMC8703828

[14] Northington FJ Chavez-Valdez R Martin LJ. Neuronal cell death in neonatal hypoxia-ischemia. Ann Neurol. (2011) 69:743–58. doi: 10.1002/ana.22419, PMID: 21520238 PMC4000313

[15] Dyall SC. Methodological issues and inconsistencies in the field of omega-3 fatty acids research. Prostaglandins Leukot Essent Fatty Acids. (2011) 85:281–5. doi: 10.1016/j.plefa.2011.04.009, PMID: 21925854

[16] Aguilar Diaz de Leon J Borges CR. Borges: evaluation of oxidative stress in biological samples using the thiobarbituric acid reactive substances assay. J Vis Exp. (2020) 12:e61122. doi: 10.3791/61122, PMID: 32478759 PMC9617585

[17] Martini S Austin T Aceti A Faldella G Corvaglia L. Free radicals and neonatal encephalopathy: mechanisms of injury, biomarkers, and antioxidant treatment perspectives. Pediatr Res. (2020) 87:823–33. doi: 10.1038/s41390-019-0639-6, PMID: 31655487

[18] Yu T Kui LQ Ming QZ. Effect of asphyxia on non-protein-bound iron and lipid peroxidation in newborn infants. Dev Med Child Neurol. (2003) 45:24–7. doi: 10.1111/j.1469-8749.2003.tb00855.x, PMID: 12549751

[19] Tharmapoopathy P Chisholm P Barlas A Varsami M Gupta N Ekitzidou G. In clinical practice, cerebral MRI in newborns is highly predictive of neurodevelopmental outcome after therapeutic hypothermia. Eur J Paediatr Neurol. (2020) 25:127–33. doi: 10.1016/j.ejpn.2019.12.018, PMID: 31882277

[20] Rutherford M Ramenghi LA Edwards AD Brocklehurst P Halliday H Levene M. Assessment of brain tissue injury after moderate hypothermia in neonates with hypoxic-ischaemic encephalopathy: a nested substudy of a randomised controlled trial. Lancet Neurol. (2010) 9:39–45. doi: 10.1016/S1474-4422(09)70295-9, PMID: 19896902 PMC2795146

[21] Bayley N. Bayley scales of infant development manual. 3rd ed. San Antonio, TX: The Psychological Corporation (2006).

[22] Bell JG Mackinlay EE Dick JR Younger I Lands B Gilhooly T. Using a fingertip whole blood sample for rapid fatty acid measurement: method validation and correlation with erythrocyte polar lipid compositions in UK subjects. Br J Nutr. (2011) 106:1408–15. doi: 10.1017/S0007114511001978, PMID: 21736805

[23] Morrison WR Smith LM. Preparation of fatty acid methyl esters and dimethylacetals from lipids with boron fluoride–methanol. J Lipid Res. (1964) 5:600–8. doi: 10.1016/S0022-2275(20)40190-7, PMID: 14221106

[24] Harris WS Von Schacky C. The Omega-3 index: a new risk factor for death from coronary heart disease? Prev Med. (2004) 39:212–20. doi: 10.1016/j.ypmed.2004.02.030, PMID: 15208005

[25] Hodson L Skeaff CM Fielding BA. Fatty acid composition of adipose tissue and blood in humans and its use as a biomarker of dietary intake. Prog Lipid Res. (2008) 47:348–80. doi: 10.1016/j.plipres.2008.03.003, PMID: 18435934

[26] Duval V Karlsson MO. Impact of omission or replacement of data below the limit of quantification on parameter estimates in a two-compartment model. Pharm Res. (2002) 19:1835–40. doi: 10.1023/a:1021441407898 12523662

[27] von Elm E Altman DG Egger M Pocock SJ Gotzsche PC Vandenbroucke JP. The strengthening the reporting of observational studies in epidemiology (STROBE) statement: guidelines for reporting observational studies. Int J Surg. (2014) 12:1495–9. doi: 10.1016/j.ijsu.2014.07.013 25046131

[28] De Rooy L Hamdallah H Dyall SC. Extremely preterm infants receiving standard care receive very low levels of arachidonic and docosahexaenoic acids. Clin Nutr. (2016) 36:1593–600. doi: 10.1016/j.clnu.2016.09.033, PMID: 27756480

[29] Brenna JT Plourde M Stark KD Jones PJ Lin YH. Best practices for the design, laboratory analysis, and reporting of trials involving fatty acids. Am J Clin Nutr. (2018) 108:211–27. doi: 10.1093/ajcn/nqy089, PMID: 29931035 PMC6084616

[30] Baack ML Puumala SE Messier SE Pritchett DK Harris WS. What is the relationship between gestational age and docosahexaenoic acid (DHA) and arachidonic acid (ARA) levels? Prostaglandins Leukot Essent Fatty Acids. (2015) 100:5–11. doi: 10.1016/j.plefa.2015.05.003, PMID: 26205427 PMC4554773

[31] Minda H Larque E Koletzko B Decsi T. Systematic review of fatty acid composition of plasma phospholipids of venous cord blood in full-term infants. Eur J Nutr. (2002) 41:125–31. doi: 10.1007/s00394-002-0366-2, PMID: 12111050

[32] van der Wurff ISM Meyer BJ de Groot RHM. Effect of omega-3 long chain polyunsaturated fatty acids (n-3 LCPUFA) supplementation on cognition in children and adolescents: a systematic literature review with a focus on n-3 LCPUFA blood values and dose of DHA and EPA. Nutrients. (2020) 12:3115. doi: 10.3390/nu12103115, PMID: 33053843 PMC7599612

[33] Poggi P Mirabella R Neri S Assirelli E Dolzani P Mariani E. Membrane fatty acid heterogeneity of leukocyte classes is altered during in vitro cultivation but can be restored with ad-hoc lipid supplementation. Lipids Health Dis. (2015) 14:165. doi: 10.1186/s12944-015-0166-3, PMID: 26703000 PMC4690394

[34] von Schacky C. Importance of EPA and DHA blood levels in brain structure and function. Nutrients. (2021) 13:1074. doi: 10.3390/nu13041074, PMID: 33806218 PMC8066148

[35] Zhang W Hu X Yang W Gao Y Chen J. Omega-3 polyunsaturated fatty acid supplementation confers long-term neuroprotection against neonatal hypoxic-ischemic brain injury through anti-inflammatory actions. Stroke. (2010) 41:2341–7. doi: 10.1161/STROKEAHA.110.586081, PMID: 20705927 PMC3021248

[36] Nessel I Khashu M Dyall SC. Effects of storage practices on long-chain polyunsaturated fatty acids and lipid peroxidation of preterm formula milk. J Hum Nutr Diet. (2021) 34:827–33. doi: 10.1111/jhn.12858, PMID: 33460485

[37] Nessel I De Rooy L Khashu M Murphy JL Dyall SC. Long-chain polyunsaturated fatty acids and lipid peroxidation products in donor human milk in the United Kingdom: results from the LIMIT 2-centre cross-sectional study. JPEN J Parenter Enteral Nutr. (2020) 44:1501–9. doi: 10.1002/jpen.1773, PMID: 32048312

[38] Gibson RA. Musings about the role dietary fats after 40 years of fatty acid research. Prostaglandins Leukot Essent Fatty Acids. (2018) 131:1–5. doi: 10.1016/j.plefa.2018.01.003, PMID: 29628045

[39] Kroger J Schulze MB. Recent insights into the relation of Delta5 desaturase and Delta6 desaturase activity to the development of type 2 diabetes. Curr Opin Lipidol. (2012) 23:4–10. doi: 10.1097/MOL.0b013e32834d2dc5, PMID: 22123669

[40] Mayneris-Perxachs J Guerendiain M Castellote AI Estruch R Covas MI Fito M. Plasma fatty acid composition, estimated desaturase activities, and their relation with the metabolic syndrome in a population at high risk of cardiovascular disease. Clin Nutr. (2014) 33:90–7. doi: 10.1016/j.clnu.2013.03.001, PMID: 23591154

[41] Svendsen K Olsen T Nordstrand Rusvik TC Ulven SM Holven KB Retterstol K. Telle-Hansen: fatty acid profile and estimated desaturase activities in whole blood are associated with metabolic health. Lipids Health Dis. (2020) 19:102. doi: 10.1186/s12944-020-01282-y, PMID: 32438926 PMC7243306

[42] Crawford MA Hassam AG Williams G. Essential fatty acids and fetal brain growth. Lancet. (1976) 1:452–3. doi: 10.1016/S0140-6736(76)91476-8, PMID: 55720

[43] Novak EM King DJ Innis SM. Low linoleic acid may facilitate Delta6 desaturase activity and docosahexaenoic acid accretion in human fetal development. Prostaglandins Leukot Essent Fatty Acids. (2012) 86:93–8. doi: 10.1016/j.plefa.2012.02.004, PMID: 22365109

[44] Nagano N Okada T Kayama K Hosono S Kitamura Y Takahashi S. Delta-6 desaturase activity during the first year of life in preterm infants. Prostaglandins Leukot Essent Fatty Acids. (2016) 115:8–11. doi: 10.1016/j.plefa.2016.09.006, PMID: 27914518

[45] Berman DR Mozurkewich E Liu Y Shangguan Y Barks JD Silverstein FS. Docosahexaenoic acid augments hypothermic neuroprotection in a neonatal rat asphyxia model. Neonatology. (2013) 104:71–8. doi: 10.1159/000351011, PMID: 23817197 PMC4721269

[46] Manual Kollareth DJ Zirpoli H Ten VS Deckelbaum RJ. Acute injection of Omega-3 triglyceride emulsion provides very similar protection as hypothermia in a neonatal mouse model of hypoxic-ischemic brain injury. Front Neurol. (2020) 11:618419. doi: 10.3389/fneur.2020.618419, PMID: 33519700 PMC7843448

[47] Andrew MJ Parr JR Montague-Johnson C Laler K Holmes J Baker B. Neurodevelopmental outcome of nutritional intervention in newborn infants at risk of neurodevelopmental impairment: the dolphin neonatal double-blind randomized controlled trial. Dev Med Child Neurol. (2018) 60:897–905. doi: 10.1111/dmcn.13914, PMID: 29806081

[48] Yan C Mao J Yao C Liu Y Yan H Jin W. Neuroprotective effects of mild hypothermia against traumatic brain injury by the involvement of the Nrf2/ARE pathway. Brain Behav. (2022) 12:e2686. doi: 10.1002/brb3.2686, PMID: 35803901 PMC9392531

[49] Huun MU Garberg HT Buonocore G Longini M Belvisi E Bazzini F. Regional differences of hypothermia on oxidative stress following hypoxia-ischemia: a study of DHA and hypothermia on brain lipid peroxidation in newborn piglets. J Perinat Med. (2018) 47:82–9. doi: 10.1515/jpm-2017-0355, PMID: 30110254

[50] Joy R Pournami F Bethou A Bhat VB Bobby Z. Effect of therapeutic hypothermia on oxidative stress and outcome in term neonates with perinatal asphyxia: a randomized controlled trial. J Trop Pediatr. (2013) 59:17–22. doi: 10.1093/tropej/fms036 22907998

[51] Brown JE Lindsay RM Riemersma RA. Linoleic acid metabolism in the spontaneously diabetic rat: delta6-desaturase activity vs. product/precursor ratios. Lipids. (2000) 35:1319–23. doi: 10.1007/s11745-000-0648-1, PMID: 11201993

[52] Morisseau C Kodani SD Kamita SG Yang J Lee KSS Hammock BD. Relative importance of soluble and microsomal epoxide hydrolases for the hydrolysis of epoxy-fatty acids in human tissues, 4993. Int J Mol Sci. (2021) 22. doi: 10.3390/ijms22094993, PMID: PMC8125816 34066758

