# Supplementary material for: Long-chain omega-3 polyunsaturated fatty acids are reduced in neonates with substantial brain injury undergoing therapeutic hypothermia after hypoxic–ischemic encephalopathy
Source: Front Neurol. 2023 Aug 30;14:1231743. doi: 10.3389/fneur.2023.1231743 (PMC10498768; doi:10.3389/fneur.2023.1231743)
Supplement: Supplementary file 1 [file Data_Sheet_1.docx]

Supplementary Material

Long-chain omega-3 polyunsaturated fatty acids are reduced in neonates with substantial brain injury undergoing therapeutic hypothermia after hypoxic-ischemic encephalopathy

Simon C. Dyall1*^✝^, Isabell Nessel^✝^, Jennine A. Sharpe, Ping Yip, Adina T. Michael-Titus, Divyen K. Shah

^✝^ These authors share first authorship

*** Correspondence:** Corresponding Author: [Simon.Dyall@roehampton.ac.uk](mailto:Simon.Dyall@roehampton.ac.uk)

**Supplementary Table S1:** Sample availability

| **Analysis** | **Controls** | **mHIE** | **sHIE+** | **sHIE-** |
| --- | --- | --- | --- | --- |
| **Fatty acids** | 10 | 8 | S1 = 10  S2 = 10  S3 = 10 | S1 = 9  S2 = 10  S3 = 10 |
| **TBARS** | - | - | S1 = 9  S2 = 9  S3 = 9 | S1 = 8  S2 = 9  S3 = 9 |
| **4-HNE** | - | - | S1 = 5  S2 = 8  S3 = 7 | S1 = 6  S2 = 7  S3 = 8 |

**Supplementary Table S2:** Feeding data for sHIE therapeutic hypothermia groups

| **Intake: day (n)** | **sHIE+**  **n=10** | **sHIE-**  **n=9*** |
| --- | --- | --- |
| Feeds commenced | d2 (2) d3 (4) d5 (2) | d2 (3) d4 (1) d5 (1) |
| Expressed breast milk | d2 (2) d3 (2) d5 (1) | d2 (2) d4 (1) d5 (1) |
| Donor human milk |  | d2 (1) |
| Formula | d3 (2) d5 (1) |  |
| Nil-by mouth | 2 | 4 |
| Parenteral nutrition | d2 (1) d3 (1) d5 (2) | d2 (2) d4 (3) |

Number in brackets indicate the number of participants

* Feeding data only available for nine infants

**Supplementary Table S3:** Table of abbreviations

| **Abbreviation** | **Definition** |
| --- | --- |
| 4-HNE | 4-Hydroxy-2-nonenal |
| ARA | Arachidonic acid (20:4n-6) |
| BGT | Basal ganglia and thalami |
| Ctr | Control |
| D5D | Δ-5 Desaturase |
| D6D | Δ-6 Desaturase |
| DHA | Docosahexaenoic acid (22:5n-3) |
| EPA | Eicosapentaenoic acid (20:5n-3) |
| HIE | Hypoxic ischemic encephalopathy |
| LA | Linoleic acid (18:2n-6) |
| MDA | Malondialdehyde |
| mHIE | Mild HIE |
| PLIC | Posterior limb of the internal capsule |
| PUFA | Polyunsaturated fatty acid |
| sHIE | Severe HIE |
| TBARS | Thiobarbituric acid reactive substances; |
| WM | White matter |
